# Long term outcome of a community-based hepatitis B awareness campaign: eight-year follow-up on linkage to care (LTC) in HBV infected individuals

**DOI:** 10.1186/s12879-019-4283-x

**Published:** 2019-07-18

**Authors:** Chul S. Hyun, Okhyun Ko, Seulgi Lee, Joseph McMenamin

**Affiliations:** 1 Center for Viral Hepatitis, 35 Van Nostrand Avenue, Englewood, NJ 07631 USA; 2 KCS Public Health and Research Center, 2 W. 32nd St. Suite 604, New York, NY 10001 USA; 3 Asian Health Services, Holy Name Medical Center, 718, Teaneck, NJ 07666 USA; 4 McMenamin Law Offices, PPLC, 10617 Falconbridge Drive, Richmond, VA 23238 USA

**Keywords:** Chronic hepatitis B, Hepatitis B virus, Health disparity, Korean Americans, Linkage-to-care, Community hepatitis B campaign

## Abstract

**Background:**

Chronic hepatitis B (CHB) is a major cause of liver-related morbidity and mortality. High HBV prevalence in immigrants and ethnic minorities and numerous barriers to healthcare access are associated with serious health disparities in the United States. Reportedly, self-awareness of HBV infection is low, suggesting a greater need for effective screening and education. Further, low levels of linkage to care (LTC) (completion of a first doctor’s visit after the diagnosis of chronic HBV infection) may be responsible for the lack of engagement over the continuum of care and for needed services.

**Methods:**

Demographics and survey data were obtained from 97 Korean American adults chronically infected with HBV, initially identified through a series of community screening events in northern New Jersey between Dec. 2009 and June 2015. Eight year follow-up on these HBV-infected individuals was obtained to determine their access to care, and thus the efficacy of a campaign to improve LTC. The participants’ self-awareness of HBV infection and other factors for LTC were also evaluated.

**Results:**

Of a total of 97 HBV-infected participants (age range 30 to 79), 74 were aware of their infections at screening. The remaining 23 had been unaware of their infections until screening. Eight years after the campaign, some 66 of these 97 individuals accessed care (LTC rate 68%). Health insurance status, presence or absence of symptoms and level of knowledge of CHB were among the most significant factors in LTC.

**Conclusion:**

A community-based hepatitis B screening and education campaign can be instrumental in prompting HBV infected individuals to access care, as demonstrated in the cumulative increase in LTC in our cohort. Despite many years of awareness of HBV infection, many are not accessing care owing to a lack of health insurance, suggesting a pressing need for advocacy and health education to improve access to affordable coverage in the Asian American population. Community efforts and strategies similar to the ones employed in the current study may serve as a model to improve the engagement of HBV-infected individuals in high risk immigrant populations.

## Background

Chronic hepatitis B (CHB) is a serious global health problem affecting 257 million people around the world, of whom 25% may eventually develop cirrhosis and cancer [1–3]. In 2015, for instance, CHB resulted in 887,000 deaths, largely from cirrhosis and hepatocellular carcinoma [1]. Notwithstanding the availability over decades of vaccines and effective antiviral medications, many people remain unprotected [3–5].

Management of CHB is also an example of health disparities in the United States, as it disproportionately affects many Asian and African immigrants and their children [4–9]. A large majority of these people have not been screened and thus are not aware of their infection or immune status. More important, many of the CHB patients who are aware of their infections are not accessing care. The current lack of access to care is attributable to numerous barriers known to impede adequate healthcare access in ethnic minority populations, resulting in poor LTC [9–12]. LTC, which refers to a first visit to a physician following the diagnosis of chronic HBV infection, is an essential step in engaging patients chronically infected with HBV and persuading them to get follow-up care. LTC involves continual monitoring of the disease and, when indicated, medical and surgical intervention.

Local community outreach CHB screening and education campaigns have been operating during the past few decades, providing an impetus for screening and vaccination in the United States. More recently, in 2011, the US Department of Health and Human Services (HHS) introduced its “Action Plan for the Prevention, Care and Treatment of Viral Hepatitis” to provide a roadmap for educating providers and communities, and to improve LTC to reduce the impact of CHB in high risk populations [13, 14]. Despite all these efforts, a majority of infected persons may remain unaware of their infections, and only a small proportion of the infected population is accessing adequate care [9–12].

From a large-scale community-based hepatitis B awareness campaign, we previously reported that a majority of the Korean Americans chronically infected with HBV were not accessing care, suggesting poor LTC in the Korean American community [11]. To determine the efficacy of the previous community outreach campaign, we assessed the proportion of the new HBV infected participants linked to care since the end of the same campaign. In addition, we examined various factors affecting LTC in these participants. The results suggest that the community education and screening campaign can be efficacious in promoting LTC, indicating a compelling need to further develop community based efforts in Hepatitis B campaigns.

## Methods

### Participants

Participants were Korean American adults chronically infected with HBV, who had previously been identified from a large-scale community-based hepatitis B screening and awareness campaign conducted in New Jersey between December 2009 and June 2015 [11]. The campaign consisted of 128 community outreach HBV screening events and educational seminars held in various locations throughout northern New Jersey. From a pool of 7157 Korean American adults, some 171 HBV infected individuals were identified between December 2009 and June 2015. During the same time period, all the HBV infected people were informed of their results and were subsequently given a list of internists and GI subspecialists within the community for consultation and follow up.

During the period between January 2017 and January 2018, we attempted to reach all 171 HBV infected individuals, of whom 129 (75.4%) were successfully contacted by phone. One hundred four of these 129 individuals agreed to participate in the telephone survey. Subsequently, 97 of these 104 participants finished a telephone survey conducted to obtain their epidemiologic and LTC status. Seven of 104 participants did not complete the survey: Of these, 4 participants thought the survey was too long and 3 others felt the information shared was too personal and sensitive. The remaining 97 participants were divided into groups A, B, C, and D based on the interval of time elapsed since the screening. Group A consisted of the participants who were screened up to 2 years before follow up, Group B screened between 2 and 4 years before follow up, and so forth (Table 3).

### Study design and survey

The current study was led by the Center for Viral Hepatitis (CVH) and the Asian Liver Center (ALC) of Holy Name Medical Center, Teaneck, NJ. Three research associates interviewed the participants: two had BS degrees in nursing, and one had an MPH. All the research associates were trained and supervised by physicians.

The participants’ demographic characteristics (sex, age, place of birth, and length of residence in the United States) were obtained at their initial screening events between December 2009 and June 2015. Once the participants were identified by name, date of birth, and HBV infection status, they were asked the following survey questions: 1). Had you been aware that you are infected with HBV, or did you discover that only through the screening campaign? 2). If before the 2009–2015 screening campaign you knew your infection status, when did you first learn it? 3.) Have you seen any physician for hepatitis B care? If so, how many times? 4). If you are seeing a physician for hepatitis B, does your provider practice medicine in a private office or in a hospital? and 5). Are you currently taking antiviral medication? In this context, LTC was defined as at least one visit to a provider for hepatitis B care. All the participants who were considered ‘linked’ to care were also tested for ALT, HBV DNA levels, and hepatoma by abdominal sonogram.

The second part of the questionnaire assessed various potential barriers hindering access to care for hepatitis B. In the first component, we evaluated a relationship between access, on one hand, and, on the other, health insurance status, English language proficiency, and family history of CHB. Second, we focused on those participants who had not accessed care and asked them to identify the most important factor that hindered their access. The answers were tabulated into four groups and summarized.

### Data collection and analysis

All the data were anonymized before they were reviewed and analyzed. A 2- tailed Fisher’s exact test was used to evaluate the differences between frequencies. The two sample z-test method was utilized to determine *p* values between the indicated sample populations.

## Results

### Demographic characteristics

The sample of 97 HBV infected subjects consisted of 55 men and 42 women with a mean age of 56.3 years. Their ages ranged between 30 and 79, with the largest cohort in the age group 50–59 followed by that of 60–69 (Table 1). All the subjects were South Korea-born Americans living in the state of New Jersey.

**Table 1:** Demographics of the HBV infected subjects

Age Group	Total	Male	Female
30–39	8	4	4
40–49	11	7	4
50–59	42	29	13
60–69	25	11	14
70–79	11	4	7
	97	55	42

### Self-awareness of infection and access to care

We evaluated how many of the HBV infected participants knew their infection status before the start of the current screening campaign. As shown in Tables 2, 74 (76%) of 97 HBV infected participants were aware of their infections before the current screening campaign began. Only 23 (34%) participants had been unaware of their HBV infections until the current campaign.

**Table 2:** Age at first time HBV diagnosis in HBsAg-seropositive participants

Interval between first diagnosis and screening (years)	HBsAg-seropositive participants (*n* = 97)	Had seen a physician for CHB at least once before screening
20 or more	35	4
10–19	22	4
2–9	17	3
At the Screening	23	0

The survey also showed that 57 of 74 participants with previous knowledge of HBV infection had known their HBV infection status for more than 10 years before being screened. Of these, 11 had seen physicians at least once after their initial diagnoses, but did not continue to follow up with physicians (Table 2).

### Efficacy of the campaign on LTC

We investigated how many of the HBV-infected participants eventually accessed care during the 8-year follow-up period after their screening. Of a total of 97 participants with various interval periods between screening and the follow-up survey, a total of 66 (68%) individuals accessed care (Table 3). Table 3 also demonstrates the number of visits to physicians participants in each group made. All of these 66 ‘linked’ participants saw physicians at least once during this interval and their visit included evaluation of ALT, HBV DNA and liver sonogram. Specifically, in group A, consisting of 26 participants followed within 1–2 years after the screening, 14 did not access care, 10 visited physicians once, and 2 saw physicians twice, with a linkage rate of 46%. Groups 2, 3, and 4, over increasing follow-up periods, showed progressively increasing linkage rates of 65, 78, and 86%, respectively. Of note, 23 of the 66 HBV infected subjects seen by physicians (35%) were started on antiviral medication.

**Table 3:** Linkage to care during the 8 year period after the screening

Groups	Interval between screening and first follow up (years)	Participants followed in the specified interval (*n* = 97)	Number of visits during the specified interval					
			0	1	2	3	4 or more	Currently on medical treatment (*n* = 23)
A	< 2	26	14	10	2	0	0	5
B	2–4	23	8	6	8	1	0	4
C	4–6	27	6	5	5	6	5	6
D	6–8	21	3	3	4	4	7	8

A total of 97 participants were divided into the groups A, B, C, and D based on the interval of time elapsed since their screening. Group A consisted of the participants who were screened up to 2 years before follow-up, group B screened between 2 and 4 years before follow up, and so forth

In further analysis of the participants’ data, 51 of the 74 (69%) individuals with previous awareness of infection accessed care while 15 of 23 (65%) first-time finders, the participants who discovered that they were chronically infected only with commencement of the campaign, accessed care (Table 4). In regard to potential patient-related factors influencing access, the survey demonstrated that health insurance coverage was closely associated with access to care. Fifty-one of 66 subjects who were accessing care had health insurance, whereas only 13 of 31 subjects not accessing care had health insurance (77% vs. 42%; *p* < 0.05). On the other hand, English language proficiency and a family history of CHB were not significantly correlated with the rate of access to care (Table 4).

**Table 4:** Health insurance and other factors in linkage-to-care

	Self-aware of infection^a^ *N* = 74			First-time finders^b^ *N* = 23		
	Access	No Access	*P* value	Access	No Access	*P* value
	51	23		15	8	
Health Insurance						
Y	40	11	*P* < .05	11	2	*P* < .05
N	11	12	*P* < .05	4	6	*P* < .05
Proficiency in English^c^						
A- Beginner to Elementary	7	7	*p* > .05	4	2	*p* > .05
B- Intermediate	19	5	*p* > .05	4	2	*p* > .05
C- Advanced	17	8	*p* > .05	5	3	*p* > .05
D- Proficient	8	3	*p* > .05	2	1	*p* > .05
Family History of CHB^d^						
Y	26	8	*p* > .05	5	2	*p* > .05
N	20	8	*p* > .05	7	4	*p* > .05
Not aware	5	7		3	2	

^a^ and ^b^ refer to the numbers of HBV infected participants who were aware and not aware, respectively, of their infection status at the time of screening events. ^c^English language levels are categorized as follows: A- make simple sentences and talk about likes and dislikes; B-take part in simple routine conversation; C- take part in lengthy conversation; D-understand the nuances of the language and engage in advanced levels of conversation. ^d^ refers to presence or absence of chronic hepatitis B in immediate family members (parents, children and siblings)

### Clinic setting of the providers

We evaluated the clinical settings where the HBV-infected participants saw the providers. Specifically, those who were currently accessing care were asked: Are you seeing a private practitioner in your community or a physician in a hospital clinic? All but five of 66 (8%) HBV infected individuals accessing care were seeing private practitioners in their communities. Two participants were seeing specialists in a university medical center-based clinic.

### Barriers to health care access

To evaluate various factors hindering HBV-infected participants from accessing care, we asked 31 participants who have not accessed care: ‘What is the most important reason you did not seek care for CHB?’ Of a total of 31 non-accessing persons, 21, of whom all but 3 did not have health insurance, considered a lack of health insurance the most important obstacle; 5 indicated that they were not seeking care because they had no symptoms; and 4 said they did not know much about the disease such that they did not feel the need to seek care. Other reasons included structural barriers such as travel and time constraints (Table 5).

**Table 5:** Reasons for not accessing care

Most important reason for not accessing care	Total (*n* = 31)
Lack of health insurance	21
Absence of Symptoms	5
Lack of knowledge on hepatitis B	4
Others	1

## Discussion

CHB remains largely undiagnosed and untreated in many parts of the world. There are 2 million HBV infected people in the United States, of whom 70% may be unaware of their infection status [9, 15]. Many individuals who are aware of their infections may not be accessing adequate care. Further, only about 10% of the CHB patients who could benefit from treatment are receiving antiviral medications [9].

### Efficacy of the hepatitis B campaign on LTC

During the past few decades, community-based hepatitis B campaigns have played an important role in educating and screening Asian American immigrants. Despite numerous such campaigns, however, studies directly examining their efficacy and sustainability with respect to LTC are lacking [16–18]. Specifically, those found through screening campaigns to be HBV infected need to be assessed for adequate LTC. Secondly, the sustainability of LTC needs to be assessed. In addition, the factors that can potentially hinder access to adequate care need to be evaluated in order to improve LTC.

The current long-term follow-up study on the 97 HBV-infected participants from our community-based hepatitis B campaign demonstrated successful care linkage and engagement. In those 66 participants who have accessed care after the screening, we found that the rate of LTC increased as the length of the follow-up period increased: 46% in 2 years, 65% in 4 years, 78% in 6 years, and 86% in 8 years (Table 3). It should be also noted that a majority of the participants followed for longer than 2 years have seen physicians more than once. For instance, in group D, 7 of 21 participants saw physicians 4 times or more during the 6–8 year follow up period. These cumulative increases in LTC seen throughout the 8-year observation may reflect the efficacy of the continually ongoing hepatitis B education and screening efforts in this community.

### Barriers to LTC

The continual monitoring and management of CHB patients are paramount in successful linkage in care of CHB. Although our study did not employ a control group, followed in the absence of a community campaign, the current and previous studies have demonstrated a significant lack of LTC in HBV infected patients [6, 9, 11, 12, 18]. For instance, it was estimated that 40% of the HBV infected subjects screened in community settings were linked to care [16, 17]. In another study involving 1231 HBV infected individuals, only 28% received additional laboratory evaluation after initial screening. [19]. Another recent study involving 1168 CHB patients reported that only one-third of the untreated patients received laboratory monitoring of HBV DNA and ALT levels at least once a year during the follow up of 728 days [20].

Given the significance of persistent monitoring in management of HBV infected patients, who can potentially put others at risk, determination of the long-term outcome of HBV screening is crucial from both individual and public health standpoints. Our finding that a majority of HBV infected participants were not engaged in any care post-diagnosis reflects a serious health access issue in immigrant and ethnic communities. It should be also stressed that in this study, despite the moderately successful outcome, as many as 31 (32%) of 97 individuals have yet to access care. Many barriers could have played important roles in this failure of adequate access. These include health insurance status, language, and other patient-related factors [6, 10, 13, 21, 22]. The current study suggests that health insurance status played a significant role in these participants’ LTC and that uninsured individuals are more likely to delay access to care than those who are covered (Table 4). Although recent nationwide expansions in insurance coverage have improved access to health care, many still lack health insurance coverage [23–25]. Further, the burden of additional expenses associated with health care utilization and borne by the individual often dissuades insureds from obtaining necessary care. The lack of knowledge of CHB and its impact on health may be another reason HBV-infected individuals avoid care, resulting in further decrease in LTC.

It has been shown in previous studies that, compared to English-proficient individuals, those with limited English proficiency had more difficulty gaining access to care [26, 27]. We, therefore, assessed the impact of language proficiency on LTC outcome. As shown in Table 4, however, patients’ English language proficiency did not appear to affect access to care in this study. These findings are congruent with the fact that the participants could find providers in the community who could speak their languages. Furthermore, family history of CHB was not associated with the rate of LTC.

In evaluating the reasons hindering LTC in the 31 participants who failed to access care, we found that the lack of health insurance was the predominant barrier (Table 5). These findings are consistent with the results of other studies that demonstrated that lack of health insurance is a major barrier in CHB care access [18, 23, 25].

Owing to the absence of symptoms and a lack of knowledge of the value of continual monitoring and possible treatment of hepatitis B, 9 of the 31 participants were not accessing care. This finding suggests that these individuals were not aware that CHB can persist, resulting in cirrhosis and/or cancer, often without obvious symptoms. These observations further point to the lack of CHB literacy. Health literacy has been directly associated with healthcare utilization [28, 29]. Thus, increasing health literacy may help improve patients’ understanding of the need for LTC, and that understanding will, in turn, lead to higher demand for access to care and thus for health insurance. Further, a patient navigator program, which has been shown to enhance access to care in hepatitis B [12, 29, 30], may be implemented to further facilitate LTC efforts.

The vigor of the search for healthcare can depend on the extent of disease in each participant, which may thus have affected the LTC in the current study. In other words, the more severe a participant’s disease, and the more he is aware of any symptom related to CHB, the more likely he may be to seek out medical care. In the absence of data on the extent of diseases in these participants, the current study does not allow us to define a true relationship between the degree of disease activity and the rate at which subjects seek medical advice.

### Self-awareness of HBV infection

It was reported in 2016 that among 257 million people worldwide chronically infected with HBV, only 27 million (10.5%) were aware of their infections [31]. Although it is noteworthy in the current study that a large proportion (76%) of the 97 HBV infected participants had previous knowledge of their infection status (Table 2), this high proportion may be an overestimate attributable to sampling. Specifically, it should be noted that the total number of infected people includes both those who are aware and those who are not aware of their infection status. Those who are aware fall into two groups: those who are currently in care (i.e., linked) and those who are not. Since those who are aware and linked did not participate in the campaign, we may be dealing with two groups: those who are aware but not linked and those who are not aware of their infection status (e.g., first-time finders). Thus, our finding that 76% of the 97 participants knew of their infections status may overestimate the true percentage of the infected people with such previous knowledge. Many of the participants aware of their infections but not in care might want to take advantage of repeat screening, thereby participating in the campaign. All these factors may also in turn end up underestimating the true percentage of the HBV infected individuals who are not aware of their infection status.

### Limitations

There are several important limitations in this study. First, the participants in the current study, consisting of 97 HBV-infected individuals, may not be representative of Korean Americans chronically infected with HBV overall. For instance, education, health and socioeconomic status of participants, all of which are important in predicting patients’ access to health care, have not been accounted for in the current study. Future studies with a larger sample accounting for participants’ health literacy and socioeconomic status would help to confirm clarify these results. Second, there may be issues with randomness of sampling as discussed above. More people with self-awareness, who are not currently seeing physicians, may have participated in the campaign as compared to those who were unaware. This would result in erroneously raising the proportion of self-aware participants relative to those who were unaware of infection. Third, there are important issues with the definition of LTC we have employed and the varying lengths of follow-up periods in this study. We have considered participants to be linked to care if they had at least one visit to a physician that included HBV DNA, ALT, and liver sonogram. This apparently has increased the percentage of linkage as the follow-up interval increased. Although some participants had visited physicians periodically once a year, many others visited randomly once every few years. Since long-term engagement for a continuum of periodic monitoring and care is critical in the care of HBV-infected individuals, some of those participants who had visited physicians less frequently than once every other year, for instance, may not be considered as accessing care as frequently as others who had visited physicians once a year. Further, while periodic assessments of ALT, DNA, and liver sonograms are essential in LTC, we have not evaluated whether each participant was regularly so tested during the follow-up periods. Lastly, we have investigated only a limited list of barriers affecting access to care: health insurance status, language, time, and knowledge of hepatitis B. The sample size is small, and more studies involving a larger number of subjects are needed to assess more accurately the interaction between and among these barriers as well as the role of other additional factors that can affect participants’ access to care in high risk populations.

## Conclusions

These findings suggest that, subject to certain limitations, a continually ongoing community-based hepatitis B campaign can successfully facilitate LTC in the Korean American community. Health care providers within the community could effectively engage HBV infected patients to access necessary services. Challenges remain to be overcome, however, to further improve and sustain access to care. The widening gap observed in this study between high awareness of infection and low level of access requires attention and effort to prompt the HBV-infected population to seek care. Barriers to LTC such as a lack of health insurance and poor health literacy are key hindering factors in LTC, and community and state-level advocacy and strategic efforts may be essential to maximize care linkage and long-term engagement in hepatitis B care.

## Data Availability

The datasets used and/or analyzed during the current study are available from the corresponding author on reasonable request.

## Abbreviations

CHB: Chronic hepatitis B
HBV: Hepatitis B virus
LTC: Linkage-to-care
